# Assessment of Mental Health of High School Students 1 Semester After COVID-19–Associated Remote Schooling Measures Were Lifted in Austria in 2021

**DOI:** 10.1001/jamanetworkopen.2021.35571

**Published:** 2021-11-22

**Authors:** Elke Humer, Rachel Dale, Paul L. Plener, Thomas Probst, Christoph Pieh

**Affiliations:** 1 Department for Psychotherapy and Biopsychosocial Health, Danube University Krems, Krems, Austria; 2 Department of Child and Adolescent Psychiatry, Medical University of Vienna, Vienna, Austria; 3 Department of Child and Adolescent Psychiatry and Psychotherapy, University of Ulm, Ulm, Germany

## Abstract

This survey study assesses whether the mental health of high school students improved 1 semester after COVID-19–associated remote schooling measures were lifted in Austria in 2021.

## Introduction

The COVID-19 pandemic and related containment efforts and restrictions (eg, lockdown measures, remote schooling, and social distancing) have been shown to be associated with impairments in mental health among adolescents.^1^ This study surveyed high school students to assess whether their mental health improved 1 semester after COVID-19–associated remote schooling and social distancing measures were lifted in Austria in 2021.

## Methods

This survey study was reviewed and approved by the Danube University Krems Ethics Committee and conducted according to the guidelines of the Declaration of Helsinki. To begin the surveys, all participants had to agree to the data protection declaration, which served as electronic informed consent. The study followed the American Association for Public Opinion Research (AAPOR) reporting guideline.

Two cross-sectional surveys were conducted with high school students aged 14 to 20 years at 2 time points to assess the effects of the COVID-19 pandemic and associated restrictions on their mental health. Both surveys assessed self-reported well-being (World Health Organization–5 Well-being Index [WHO-5]^2^), depressive symptoms (Patient Health Questionnaire–9 [PHQ-9]^3^), anxiety symptoms (Generalized Anxiety Disorder–7 Screener [GAD-7]^4^), sleep quality (Insomnia Severity Index [ISI]^5^), and perceived stress level (Perceived Stress Scale–10 [PSS-10]^6^). The first survey was conducted after 1 semester of almost exclusively remote schooling, with online data collection from February 3 to 28, 2021 (time 1). The Austrian Federal Ministry of Education, Science, and Research informed and invited all schools to participate. The second survey was conducted after 1 semester of school reopenings, with online data collection from June 19 to July 2, 2021 (time 2). School representatives emailed the online link for the second survey to students and invited them to participate via social media platforms.

A matched-pairs analysis according to age, sex, region, school type, and migration status (ie, whether participants or both parents were born abroad) was computed using the MatchIt package in R software (eMethods in the Supplement). Statistical significance was set at .05 (2-tailed). Effect sizes are shown as Cohen *d*.

## Results

A total of 3052 adolescents participated in the first survey, and 2139 (70.1%) were female. Their mean (SD) age was 16.5 (1.4) years, and 508 (16.6%) had a migration background. A total of 720 adolescents participated in the second survey, and 568 (78.9%) were female. Their mean (SD) age was 16.3 (1.3) years, and 91 (12.6%) had a migration background. In total, 545 adolescents who participated at time 2 could be matched with participants at time 1 according to age, sex, region, education, and migration status.

Matched-sample analyses showed a mean (SD) change from time 1 to time 2 for participant self-ratings of well-being (WHO-5: 32.7 [19.1] to 42.4 [21.5]), depressive symptoms (PHQ-9: 14.3 [6.3] to 11.6 [6.6]), anxiety symptoms (GAD-7: 11.9 [5.1] to 9.9 [5.3]), insomnia (ISI: 11.8 [5.5] to 10.0 [6.0]), and stress levels (PSS-10: 25.8 [6.7] to 21.1 [7.8]) (all *P* < .001). Table 1 summarizes the results of the mental health measurements. Table 2 shows the statistical results and cutoff values by sex and time point. Although female students had improved scores for all variables investigated at time 2 compared with time 1 (*P* < .001), male students had improved scores for well-being, anxiety, and stress levels at time 2 compared with time 1 (all *P* < .01). Effect sizes ranged from 0.10 to 0.94 (Table 2).

**Table 1.** Measures of Psychological Health by Sex and Time Point (Matched Sample)

Measure	Time 1^a^			Time 2^b^		
	Total (n = 545)^c^	Female respondents (n = 473)	Male respondents (n = 68)	Total (n = 545)^c^	Female respondents (n = 432)	Male respondents (n = 105)
**WHO-5**						
Score, mean (SD)	32.7 (19.1)	31.7 (18.6)	40.5 (20.3)	42.4 (21.5)	40.6 (20.8)	50.9 (22.2)
**PHQ-9**						
Score, mean (SD)	14.3 (6.3)	14.9 (6.2)	10.2 (6.2)	11.6 (6.6)	12.2 (6.5)	8.5 (6.0)
≥11, No. (%)	381 (69.9)	347 (73.4)	30 (44.1)	270 (49.5)	232 (53.7)	31 (29.5)
**GAD-7**						
Score, mean (SD)	11.9 (5.1)	12.4 (4.9)	8.7 (5.1)	9.9 (5.3)	9.5 (5.2)	6.5 (4.8)
≥11, No. (%)	318 (58.4)	294 (62.2)	20 (29.4)	202 (37.1)	178 (41.2)	19 (18.1)
**ISI**						
Score, mean (SD)	11.8 (5.5)	12.2 (5.4)	9.2 (5.4)	10.0 (6.0)	10.5 (5.9)	7.8 (6.0)
≥15, No. (%)	163 (29.9)	152 (32.1)	9 (13.2)	110 (20.2)	92 (21.3)	16 (15.2)
**PSS-10**						
Score, mean (SD)	25.8 (6.7)	26.4 (6.2)	21.0 (8.2)	21.1 (7.8)	22.0 (7.5)	16.9 (7.5)
≥27, No. (%)	260 (47.7)	239 (50.5)	18 (26.5)	139 (25.5)	126 (29.2)	9 (8.6)

Abbreviations: GAD-7, Generalized Anxiety Disorder–7 Screener; ISI, Insomnia Severity Index; PHQ-9, Patient Health Questionnaire–9; PSS-10, Perceived Stress Scale–10; WHO-5, World Health Organization–5 Well-being Index.

^a^ Time 1 indicates the period from February 3 to 28, 2021, 1 semester after almost exclusively remote schooling.

^b^ Time 2 indicates the period from June 19 to July 2, 2021, after 1 semester of school reopenings.

^c^ The total number of participants for time 1 and time 2 includes individuals whose gender identity or gender expression does not conform to socially defined male or female gender norms (n = 4 at time 1 and n = 8 at time 2).

**Table 2.** Statistical Results for the Comparison Between Time 1 and Time 2 (Matched-Sample Analyses)^a^

Measure	Total			Female respondents			Male respondents		
	Statistic (95% CI)	*P* value^b^	Cohen *d* (95% CI)^c^	Statistic (95% CI)	*P* value	Cohen *d* (95% CI)	Statistic (95% CI)	*P* value	Cohen *d* (95% CI)
**WHO-5**									
Mean score	t_1087_ = 7.54 (6.92 to 11.79)	<.001	0.22 (0.17 to 0.28)	t_902_ = 6.22 (5.66 to 10.88)	<.001	0.20 (0.14 to 0.27)	t_170_ = 2.96 (3.32 to 16.61)	.004	0.22 (0.07 to 0.37)
**PHQ-9**									
Mean score	t_1087_ = −6.67 (−3.42 to −1.86)	<.001	−0.20 (−0.26 to −0.14)	t_902_ = −5.78 (−3.3 to −1.63)	<.001	−0.19 (−0.26 to −0.13)	t_170_ = −1.75 (−3.57 to 0.21)	.08	−0.13 (−0.29 to 0.02)
Cutoff ≥11	z_1089_ = −6.65 (−1.1 to −0.6)	<.001	−0.43 (−0.55 to −0.3)	z_904_ = −5.76 (−1.11 to −0.55)	<.001	−0.42 (−0.56 to −0.27)	z_172_ = −1.9 (−1.27 to 0.02)	.06	−0.3 (−0.62 to 0.01)
**GAD-7**									
Mean score	t_1087_ = −9.22 (−3.54 to −2.3)	<.001	−0.27 (−0.33 to −0.21)	t_902_ = −8.16 (−3.46 to −2.12)	<.001	−0.27 (−0.33 to −0.2)	t_170_ = −2.75 (−3.64 to −0.6)	.007	−0.21 (−0.36 to −0.06)
Cutoff ≥11	z_1089_ = −6.79 (−1.1 to −0.61)	<.001	−0.43 (−0.55 to −0.3)	z_904_ = −5.88 (−1.08 to −0.54)	<.001	−0.41 (−0.54 to −0.27)	z_172_ = −1.69 (−1.36 to 0.1)	.09	−0.31 (−0.66 to 0.05)
**ISI**									
Mean score	t_1087_ = −5.09 (−2.47 to −1.1)	<.001	−0.15 (−0.21 to −0.09)	t_902_ = −4.08 (−2.3 to −0.81)	<.001	−0.14 (−0.2 to −0.07)	t_170_ = −1.57 (−3.21 to 0.37)	.12	−0.12 (−0.27 to 0.03)
Cutoff ≥15	z_1089_ = −3.56 (−0.79 to −0.23)	<.001	−0.26 (−0.4 to −0.12)	z_904_ = −3.41 (−0.84 to −0.23)	<.001	−0.27 (−0.42 to −0.11)	z_172_ = −0.44 (−0.67 to 1.12)	.66	0.10 (−0.33 to 0.55)
**PSS-10**									
Mean score	t_1087_ = −10.32 (−5.43 to −3.7)	<.001	−0.30 (−0.36 to −0.24)	t_902_ = −8.98 (−5.02 to −3.22)	<.001	−0.29 (−0.35 to −0.23)	t_170_ = −3.31 (−6.43 to −1.63)	.001	−0.25 (−0.39 to −0.1)
Cutoff ≥27	z_1089_ = −6.80 (−2.17 to −1.21)	<.001	−0.83 (−1.09 to −0.6)	z_904_ = −5.58 (−2.6 to −1.26)	<.001	−0.94 (−1.3 to −0.63)	z_172_ = −2.49 (−1.78 to −0.23)	.01	−0.48 (−0.87 to −0.11)

Abbreviations: GAD-7, Generalized Anxiety Disorder–7 Screener; ISI, Insomnia Severity Index; PHQ-9, Patient Health Questionnaire–9; PSS-10, Perceived Stress Scale–10; WHO-5, World Health Organization–5 Well-being Index.

^a^ Time 1 indicates the period from February 3 to February 28, 2021, 1 semester after almost exclusively remote schooling; time 2 indicates the period from June 19 to July 2, 2021, after 1 semester of school reopenings.

^b^ *P* values are 2-tailed.

^c^ Cohen *d* was calculated as an effect size measure for differences (small effect size: 0.2 to 0.5; medium effect size: 0.5 to 0.8; and large effect size: >0.8).

## Discussion

These study results suggest an improvement in the mental health burden of adolescents 1 semester after school reopenings and social distancing measures were reduced. There are several possible explanations for these findings. Apart from school reopenings, the forthcoming start of the summer holidays, loosening of other public health restrictions, increased vaccination rates, or decreased rates of COVID-19 infection could influence the mental health of adolescents. However, scores on assessments of current mental health indicators, such as those used in this study, remain substantially higher among this age group than before the COVID-19 pandemic.

This study has several limitations, including its cross-sectional nature, its small sample size, the exclusive use of self-rating instruments, and the possibility of a self-selection bias attributable to the online implementation of the study. In addition, the male sample was smaller than the female sample, and notable effects are more probable in larger samples.

In summary, the results of this study suggest that school reopenings and reduced social distancing measures correlate with improved mental health measures among high school students. Further studies should validate these results. Because of the high prevalence of mental health symptoms among adolescents, psychological support should be offered promptly.

## Notes

Supplement.
eMethods.
