# Supplementary material for: Assessment of Mental Health of High School Students 1 Semester After COVID-19–Associated Remote Schooling Measures Were Lifted in Austria in 2021
Source: JAMA Netw Open. 2021 Nov 22;4(11):e2135571. doi: 10.1001/jamanetworkopen.2021.35571 (PMC8609406; doi:10.1001/jamanetworkopen.2021.35571)
Supplement: Supplement. — eMethods. [file jamanetwopen-e2135571-s001.pdf]

Supplementary Online Content

Humer E, Dale R, Plener PL, Probst T, Pieh C. Assessment of mental health of high school students 1 semester after COVID-19–associated remote schooling measures were lifted in Austria in 2021. *JAMA Netw Open*. 2021;4(11):e2135571. doi:10.1001/jamanetworkopen.2021.35571

eMethods.

This supplementary material has been provided by the authors to give readers additional information about their work.

© 2021 Humer E et al. *JAMA Network Open*.

eMethods.

1. COVID-19 restrictions in Austria

From October 2020 to February 2021 high school took mainly place in remote schooling. During this time, no team or indoor sport was allowed. In November 2020 and from December 2020 until January 2021 one was allowed to leave the house only in a few exceptions, such as necessary purchases, assisting other people or activities outside, alone or with people living in the same household. Starting on February 8, 2021 the strict lockdown was lifted and high schools, retail shops, service providers, museums, parks, zoos etc. were allowed to reopen with protective measures such as wearing FFP2 face masks. Classes were reopened in a shift system and extended protective measures such as weekly COVID-19 tests and mandatory FFP2-mask wearing. After six months of complete shutdown of gastronomy, hotels, events, etc., they reopened from May 19, 2021. The reopening was integrated with the “3G rule”, referring to the need to prove that one has been vaccinated, tested or recovered from COVID-19, to visit restaurants, hotels, take part in events, etc.

2. Measures

To assess the impact of the COVID-19 pandemic and associated restrictions on mental health, internationally established, comparable, and validated instruments previously used in adolescents were used:

2.1. Well-being (WHO-5)

The WHO-5[1] comprises five self-rating items rating well-being on a six-point Likert scale. The total score was multiplied by four, resulting in a range from 0 (absence of well-being) to 100 (maximal well-being). The German version of the WHO-5 has been validated in adolescents, showing good psychometric properties.[2]

2.2. Depressive symptoms (PHQ-9)

Depressive symptoms were assessed with the depression module of the Patient Health Questionnaire-9 (PHQ-9).[3] This questionnaire comprises nine self-rating items on a four-point scale ranging from 0 (“not at all”) to 3 (“nearly every day”) with a sum score from 0 to 27, with higher scores indicating more severe

© 2021 Humer E et al. *JAMA Network Open*.

depressive symptoms. The German version of the PHQ-9 is validated for adolescents.[4] Clinically relevant depression was defined as cut-off score of ≥11 points, as this cut-off has been recommended for detecting major depression in adolescents.[5]

2.3. Anxiety symptoms (GAD-7)

Anxiety symptoms were measured with the German version of the Generalized Anxiety Disorder-7 Screener (GAD-7).[6] The GAD-7 assesses anxiety with 7 self-rating items on a four-point scale, from 0 to 3 with a sum score from 0 to 21, with higher scores indicating higher anxiety levels. As suggested adolescents,[7] a cut-off score of ≥11 was used to define clinically relevant (moderate) anxiety.

2.4. Sleep quality (ISI)

The German version of the Insomnia Severity Index (ISI) is a validated screening instrument for measuring sleep quality and insomnia in adolescents.[8] The ISI comprises 7 self-reported items on a four-point scale ranging from 0 to 4, resulting in a sum score from 0 to 28, with higher scores indicating worse sleep quality. The recommended cut-off score for clinically relevant insomnia (moderate severity) is ≥ 15 points.[9]

2.5. Stress level (PSS-10)

Perceived stress level was measured with the Perceived Stress Scale-10 (PSS-10), a validated questionnaire comprising 10 self-rated items ranging from 0 to 4 (maximum score 40), with higher scores indicating higher stress levels.[10] The German version is validated for adolescents. A cut-off ≥ 27 points indicates high stress.[11]

2.6. Migration status

Students were asked whether they or both parents were born abroad, to assess their migration status.

3. Statistical analyses

A matched pairs analysis according to age, sex, region, school type and migration status was computed using the MatchIt package[12] of R version 4.1.0. We assigned a propensity score to each participant representing the probability of belonging to one of the two groups, given a vector of observed covariates.[13]

© 2021 Humer E et al. *JAMA Network Open*.

After systematic examination of multiple matching techniques, the 'optimal' method was chosen because it produced the best matching, as assessed by the percent balance improvement, jitter plots and histograms. The matching process creates a new 'propensity score' variable which must be included as a covariate in all future analyses.[14] Therefore, after matching, the two time points were compared in the mental health outcome measures using linear models with time point and propensity score as fixed factors. For assessing the likelihood of being over the scale specific cut-offs for clinically relevant symptoms, general linear models were used with the binomial family. Data were analyzed from July 3 to July 28, 2021.

4. Completion rate

Following the American Association for Public Opinion Research (AAPOR) reporting guidelines for non-probability online samples,[15] we report that 79.5% of those who clicked on the survey link completed the entire survey, resulting in a 79.5% participation rate (i.e., completion rate).[16]

References

1. Brähler E, Mühlan H, Albani C, Schmidt S. Teststatistische Prüfung und Normierung der Deutschen Versionen des EUROHIS-QOL Lebensqualität-index und des WHO-5 Wohlbefindens-index. *Diagnostica*. 2007;53(2):83-96. doi:10.1026/0012-1924.53.2.83

2. Allgaier AK, Pietsch K, Frühe B, Prast E, Sigl-Glöckner J, Schulte-Körne G. Depression in pediatric care: Is the WHO-Five Well-Being Index a valid screening instrument for children and adolescents? *Gen Hosp Psychiatry*. 2012;34(3):234-241. doi:10.1016/j.genhosppsych.2012.01.007

3. Spitzer RL, Kroenke K, Williams JBW. Validation and utility of a self-report version of PRIME-MD: The PHQ primary care study. *JAMA*. 1999;282(18):1737-1744. doi:10.1001/jama.282.18.1737

4. Allgaier AK, Pietsch K, Frühe B, Sigl-Glöckner J, Schulte-Körne G. Screening for depression in adolescents: Validity of the Patient Health Questionnaire in pediatric care. *Depress Anxiety*. 2012;29(10):906-913. doi:10.1002/da.21971

© 2021 Humer E et al. *JAMA Network Open*.

5. Richardson LP, McCauley E, Grossman DC, et al. Evaluation of the Patient Health Questionnaire-9 item for detecting major depression among adolescents. *Pediatrics*. 2010;126(6):1117-1123. doi:10.1542/peds.2010-0852

6. Löwe B, Decker O, Müller S, et al. Validation and standardization of the Generalized Anxiety Disorder Screener (GAD-7) in the general population. *Med Care*. 2008;46(3):266-274. doi:10.1097/MLR.0b013e318160d093

7. Mossman SA, Luft MJ, Schroeder HK, et al. The Generalized Anxiety Disorder 7-item scale in adolescents with generalized anxiety disorder: Signal detection and validation. *Ann Clin Psychiatry*. 2017;29(4):227-234A.

8. Gerber M, Lang C, Lemola S, et al. Validation of the German version of the insomnia severity index in adolescents, young adults and adult workers: Results from three cross-sectional studies. *BMC Psychiatry*. 2016;16(1):174. doi:10.1186/s12888-016-0876-8

9. Morin CM, Belleville G, Bélanger L, Ivers H. The insomnia severity index: Psychometric indicators to detect insomnia cases and evaluate treatment response. *Sleep*. 2011;34(5):601-608. doi:10.1093/sleep/34.5.601

10. Cohen S, Kamarck T, Mermelstein R. A global measure of perceived stress. *J Health Soc Behav*. 1983;24:385-396. doi:10.2307/2136404

11. Klein EM, Brähler E, Dreier M, et al. The German version of the perceived stress scale - psychometric characteristics in a representative German community sample. *BMC Psychiatry*. 2016;16:159. doi:10.1186/S1288-016-0875-9

12. Ho DE, Imai K, King G, Stuart EA. MatchIt : Nonparametric preprocessing for parametric causal inference. *J Stat Soft*. 2011;42(8). doi:10.18637/jss.v042.i08

13. Rosenbaum PR, Rubin DB. The central role of the propensity score in observational studies for causal effects. *Biometrika.* 1983;70(1):41–55. doi:10.1093/biomet/70.1.41

14. Bartak A, Spreeuwenberg MD, Andrea H, et al. The use of propensity score methods in psychotherapy research. *Psychother Psychosom.* 2009; 78(1): 26-34. doi:0.1159/000162298

© 2021 Humer E et al. *JAMA Network Open*.

15. American Association for Public Opinion Research. *Standard Definitions: Final Dispositions of Case Codes and Outcome Rates for Surveys*. 9th ed. American Association for Public Opinion Research; 2016.

16. Callegaro M, DiSogra, C. Computing response metrics for online panels. *Public Opinion Q.* 2008;72(5): 1008-1032. doi:10.1093/poq/nfn065

© 2021 Humer E et al. *JAMA Network Open*.
